# Lack of iron, zinc, and vitamins as a contributor to the etiology of atopic diseases

**DOI:** 10.3389/fnut.2022.1032481

**Published:** 2023-01-09

**Authors:** Diego G. Peroni, Karin Hufnagl, Pasquale Comberiati, Franziska Roth-Walter

**Affiliations:** ^1^Section of Paediatrics, Department of Clinical and Experimental Medicine, University of Pisa, Pisa, Italy; ^2^Comparative Medicine, The Interuniversity Messerli Research Institute of the University of Veterinary Medicine Vienna, Medical University of Vienna and University of Vienna, Vienna, Austria; ^3^Institute of Pathophysiology and Allergy Research, Center of Pathophysiology, Infectiology and Immunology, Medical University of Vienna, Vienna, Austria

**Keywords:** iron, vitamin A, vitamin D, inflammation, micronutritional deficiencies, anemia, atopic diseases, children

## Abstract

Micronutritional deficiencies are common in atopic children suffering from atopic dermatitis, food allergy, rhinitis, and asthma. A lack of iron, in particular, may impact immune activation with prolonged deficiencies of iron, zinc, vitamin A, and vitamin D associated with a Th2 signature, maturation of macrophages and dendritic cells (DCs), and the generation of IgE antibodies. In contrast, the sufficiency of these micronutrients establishes immune resilience, promotion of regulatory cells, and tolerance induction. As micronutritional deficiencies mimic an infection, the body’s innate response is to limit access to these nutrients and also impede their dietary uptake. Here, we summarize our current understanding of the physiological function of iron, zinc, and vitamins A and D in relation to immune cells and the clinical consequences of deficiencies in these important nutrients, especially in the perinatal period. Improved dietary uptake of iron is achieved by vitamin C, vitamin A, and whey compounds, whereas zinc bioavailability improves through citrates and proteins. The addition of oil is essential for the dietary uptake of beta-carotene and vitamin D. As for vitamin D, the major source comes *via* sun exposure and only a small amount is consumed *via* diet, which should be factored into clinical nutritional studies. We summarize the prevalence of micronutritional deficiencies of iron, zinc, and vitamins in the pediatric population as well as nutritional intervention studies on atopic diseases with whole food, food components, and micronutrients. Dietary uptake *via* the lymphatic route seems promising and is associated with a lower atopy risk and symptom amelioration. This review provides useful information for clinical studies and concludes/emphasizes that a healthy, varied diet containing dairy products, fish, nuts, fruits, and vegetables as well as supplementing foods or supplementation with micronutrients as needed is essential to combat the atopic march.

## 1. Introduction

Micronutrients are minerals and vitamins, which are vital in very small amounts for the body’s health. Deficiencies here can cause severe and even life-threatening conditions, but can also lead to clinically less noticeable impairments in energy levels, mental clarity, and overall performance, as well as an increased risk for other diseases, particularly, immune-mediated ones.

There is an intricate network between nutrients and our immune system as humans (and vertebrates, in general) have developed numerous strategies termed nutritional immunity (1) to starve invading pathogens by withholding and depriving them of micronutrients. However, for proper growth and function, the immune cells need micronutrients (e.g., iron, zinc, selenium, copper, folate, and vitamins A/D/C) (2).

Indeed, a lack of these micronutrients may signal danger to the immune system and leads to their priming/activation which, if mild, can even reduce the risk of infections: mild iron deficiency appears to be protective against the development of parasitic infection (e.g., *Plasmodium falciparum*) (3–5) (ISRCTN32849447). In contrast, when the host’s strategies to withhold and deprive these pathogens of micronutrients fails, these pathogens, together with the defense strategies to withhold and block micronutrient uptake, may aggravate the situation and lead to anemia and chronic inflammation.

An adequate nutritional balance is, thus, of utmost importance when growing up with the nutritional status already passed at birth from the mother to the child. Especially, during the first months of life, nutrition *via* breast milk is considered superior to milk formulas as it includes nutrients and essential immune factors for the growth and development of infants until 1 year. However, 5 months after birth, the nutrient level in breast milk starts to diminish in minerals, proteins, and vitamins and so the introduction of food should commence in conjunction with breastfeeding (6), as breast milk alone is no longer sufficient to meet the nutritional requirements in terms of energy and micronutrients (iron and zinc) after 6 months of life.

Moreover, the nutritional quality of breast milk differs significantly with undernourishment, reducing the levels of several micronutrients including vitamin A and all B vitamins except folate, iodine, and selenium (7). The consumption of a limited range of food or avoiding allergenic food, which is usually rich in micronutrients, can further diminish the micronutritional content. In addition, in atopic mothers, the presence of low-grade inflammation further hampers the normal dietary uptake of these essential micronutrients.

In a Danish study on atopic mothers (8), the breast milk of atopic women was found to contain lower amounts of vitamin D (9) and vitamin C (10) and have a modified oligosaccharide (11) and fatty acid profile (12–17), with the lipidic composition being closely associated with the diet and time of sampling. Although not assessed so far, it can be assumed that the breast milk of atopic women contains lower levels of vitamin A (retinol), iron, B vitamins, iodine, and selenium (18–21) (NCT00164736, NCT00164762).

Infants with atopic diseases are also likely to be affected by dietary restriction which further emphasizes the increased risk of nutritional inadequacies that may contribute to the development of allergic diseases.

Although this review focuses on micronutritional deficiencies of iron, zinc, as well as vitamins A and D as the predominant driver of atopic diseases, it is important to note that an excess amount of these micronutrients will also cause inflammation though *via* different mechanisms and may, thereby, also contribute to the pathogenesis of atopic diseases (22–25) (NCT00168597, NCT01779180).

In this review, special attention will be given to the micronutrients of iron, zinc, vitamin A, and vitamin D as key elements and modulators of immune cells. The main characteristics of these micronutrients and the basic mechanisms of nutrient uptake in healthy and inflamed conditions will be discussed, and evidence will be provided on the effects on immune cells. Clinical evidence for micronutritional deficiencies in atopic children and the impact of the diet and dietary elements on the disease course are discussed. Importantly, many of these deficiencies can be prevented through nutritional education and the consumption of a healthy, varied diet, as well as by fortifying and supplementing foods or direct supplementation as needed. The review also provides useful information for ensuring the bioavailability of these precious micronutrients, when deficiencies and inflammation are already present.

## 2. Micronutrients

### 2.1. Iron

Iron deficiency is the most widespread nutritional disorder worldwide and is a public health problem in both industrialized and non-industrialized countries. The assessment of iron deficiency is complicated by the fact that iron parameters such as ferritin, transferrin saturation, and zinc protoporphyrin are affected in any infectious or inflammatory process, with the presence of (low-grade) inflammation leading to an underestimation of iron deficiency.

Iron deficiency is the result of a long-term negative iron balance; in its more severe manifestations, iron deficiency leads to anemia, which indeed represents an extreme form and is defined as a low hemoglobin concentration in the blood. The hemoglobin thresholds that indicate anemia vary according to physiological status (e.g., age and sex) and have been established by WHO for different population groups (26), e.g., in children, the threshold is <110 g/L for the age of 6–59 months.

Iron deficiency can also be “functional” in its nature, in which ferritin levels are usually within normal limits. Here, the iron supply and incorporation into erythroid precursors are insufficient, despite the presence of apparently adequate body iron stores. In this case, iron is present, but metabolically inactive, i.e., meaning it is stored within ferritin, in reticuloendothelial cells, which consist primarily of monocytes and macrophages, and is unavailable for immediate use (27) (H15-00721). This “functional iron deficiency,” blocking metabolic active iron, is seen in subjects with infectious, inflammatory, and malignant diseases, and is a major component of the anemia of chronic disease. However, functional iron deficiency also occurs in high-performance athletes due to exercise-induced inflammation (28) and in obese people because of the presence of low-grade inflammation.

It is, therefore, essential not to interchange the terms “iron deficiency” and “iron-deficiency anemia” as they describe different conditions.

#### 2.1.1. Iron uptake under inflammation: A real problem

The discovery of the iron regulatory peptide, hepcidin, a 25-amino acid peptide synthesized in the liver, in regulating iron homeostasis in 2001 completely revolutionized our understanding of iron disorders (29). Hepcidin is upregulated in the setting of inflammation and cancer, resulting in its increased synthesis in the liver stimulated by cytokines of which interleukin 6 is the most important. By degrading ferroportin, which enables exporting iron into circulation, hepcidin decreases iron absorption from the gastrointestinal tract and decreases the accessibility of iron stored in macrophages. The blocking of iron absorption and mobilization is a critical clinical finding, which is associated with a worsened prognosis and outcome in chronic diseases such as congestive heart failure (30, 31), chronic kidney diseases (32–34) (NCT03029208, NCT02940860, and NCT01864161), autoimmune diseases (35–39), inflammatory bowel disease (40, 41), cancer (42–44), atopic diseases (45, 46) (NCT03815981 and NCT03816800), and even in obesity (47–50) (NCT00030238). Importantly, in iron-deficient children who are not anemic, inflammation is a common finding (51).

**FIGURE 1:**
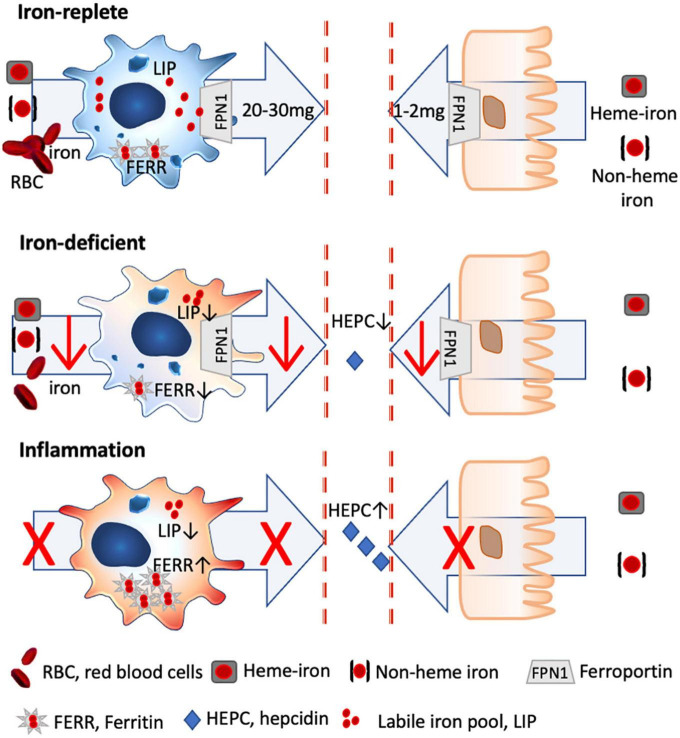
Iron homeostasis in steady state and inflammatory conditions. Only 2 mg of dietary iron is absorbed *via* the gut daily, with the bioavailability of heme iron being about 5 × greater than non-heme iron. In contrast, 20–30 mg of iron is recycled iron from senescent red blood cells by splenic macrophages, which are characterized by a high iron turnover rate with a large labile iron pool (LIP) and low ferritin levels (FERR). Under iron-deficient conditions, less iron is absorbed *via* the gut and macrophages shift toward a more activated status, as iron through-put and thus metabolically active iron is low (low LIP), while FERR is also low. Under inflammatory conditions, hepcidin is excreted which degrades ferroportin, with the result that dietary iron uptake is hampered. Likewise, macrophages stop further iron acquisition and export and store the available iron into FERR, with the result that inflammatory macrophages have a low LIP, while FERR is increased.

There are multiple dietary iron uptake mechanisms: uptake of heme iron (meat and fish) is receptor-mediated and about five times more efficient than the uptake of none-heme iron (plants, grains, and legumes), which must be reduced to ferrous iron before uptake and is facilitated by vitamin C (27) (Figure 1). In children, iron deficiency is particularly important as it can affect not only growth but also the lung (52, 53) (IRB No. 2017-04-049), and also the small intestinal function is impaired. Iron-deficient children also have poorer performance rates and its association with impaired cognitive development (attention, sensory perception, emotions, and intelligence) is well recognized. Importantly, functional iron deficiency is present in obese children, which impedes iron absorption, despite similar dietary iron intake (54). As normal iron uptake is impaired in situations of inflammation, newer oral iron formulations such as ferrous iron encapsulated in a phospholipid bilayer (55) or liposomal iron and ferric iron in starchlike vesicles (56) have been developed to circumvent hepcidin-mediated blockage of iron absorption by using the lymphatic route for iron uptake (57).

#### 2.1.2. Prevalence of iron deficiency in atopic children

Epidemiological studies conducted in the US (58) and Korea (59) have attested that children with atopic diseases such as atopic dermatitis (60), wheeze, and allergic rhinitis/conjunctivitis are up to 8 times more likely to be anemic compared to children without any allergies. In addition, smaller studies have reported a high prevalence of iron and zinc deficiencies in children with atopic dermatitis (61), with low serum iron associated with lower lung function (52).

Contributing factors listed for greater anemic risk, apart from food avoidance, were chronic inflammation and the use of systemic immunosuppressive medications. Indeed, an inadequate iron intake in the weaning period, when the child’s micronutrient needs can no longer be met by breast milk alone, has been reported in infants with atopic dermatitis (6), emphasizing that food introduction after 6 months is essential for proper growth, as the nutrient levels of iron in breast milk start to decrease after 5 months (62) (NCT01444261, NCT00841061, and NCT00760890). Similar lower iron intake has been described in children with atopic dermatitis (63, 64).

Also, a greater prevalence of helminth and protozoa infections has been reported in children with atopic dermatitis and wheeze (65–69) (ISRCTN41239086), which was associated with symptoms (66, 70–72). In these cases, it was shown that deworming strategies decreased the risk of asthma (73) but not of other atopic diseases.

Conversely, also children with anemic diseases such as sickle cell disease (74, 75) or beta-thalassemia major (76–79) (CGMF- 201801200B0) are more likely to have atopic diseases and suffer from asthma (80, 81), indicating that iron itself is involved in the etiology of atopic diseases. Adults and children diagnosed with atopic dermatitis are also at greater risk of developing associated autoimmune diseases such as Crohn’s disease, pernicious anemia, autoimmune hypothyroidism, rheumatoid and psoriatic arthritis, vitiligo, and alopecia areata (82). Similarly, also other allergic diseases such as rhinitis/conjunctivitis and asthma are associated with a greater incidence of autoimmune diseases (83), emphasizing the close link between iron and our immune system.

As the nutritional state of the mother is passed to the child, also maternal iron status impacts the risk of allergies in children. The Avon Longitudinal Study of Parents and Children associated reduced umbilical cord iron levels with childhood wheeze and eczema (84), whereas several studies associated a reduced maternal iron status during pregnancy adversely with childhood wheeze, lung function, and atopic sensitization (85–88) (NCT03408275, NCT03408275, NCT01647399, and NCT01308112). In the same line but vice versa, the study by Fortes et al. reported that the children of women who were supplemented with iron and folic acid during pregnancy had a fourfold reduced risk of developing atopic dermatitis (89).

To summarize, there is compelling evidence that children suffering from atopic diseases lack iron with all its detrimental implications.

#### 2.1.3. Macrophages: Immune sentinels and the central hub for iron distribution

To understand why the lack of iron, in particular, is associated with atopic diseases, one must delve into iron physiology, described in detail elsewhere (27), and its association with our immune system.

Briefly, about 1–2 mg of iron is absorbed daily through the intestine, while 20–30 mg of iron is recycled predominantly by splenic macrophages from senescent red blood cells (90). This means that the primary cells dealing and distributing iron in our body are macrophages, and any change in the iron levels and status will thus directly affect these cells.

Macrophages are crucial for their surveillance role in pathogen recognition and for their homeostatic function of clearing the surroundings from apoptotic and senescent cells *via* phagocytosis. In recent years, another function of macrophages has been well recognized, namely, that of acting both as a sensor for the nutrient demand of the surrounding tissues and as a supplier of iron (91).

Although there is a broad range of macrophage subtypes, the prototypical pro-inflammatory and anti-inflammatory macrophages can be distinguished by their iron-handling features. Pro-inflammatory M1 macrophages neither partake in iron sequestration nor export, and intracellularly their labile and metabolic active iron levels are low, as the available iron is entrapped within ferritin, making it inaccessible for pathogens, but also nutritional supply (92). In contrast, anti-inflammatory M2 macrophages that usually display a high expression of CD163, the hemoglobin/haptoglobin receptor essential for heme iron import, possess a large labile iron pool, which represents the metabolic active iron within the cell, and only a small amount of iron is stored within ferritin.

Importantly, the by-default anti-inflammatory phenotype of macrophages changes under iron-deficient conditions. In the absence of iron, less iron is supplied to the macrophage, which means that iron turnover is lower, resulting in a decline of metabolically active iron. Consequently, the classical characteristics of the anti-inflammatory macrophage with a large labile iron pool and a high turnover rate are changed toward a more pro-inflammatory phenotype (27).

Nutritional iron deficiency has been implicated in low-grade inflammation (93) (NCT01088958), with a more pro-inflammatory state of the monocytic cells being reported in children (94) and infants (95) with iron deficiency (Figure 1).

#### 2.1.4. Iron deficiency in B cells, T cells, and mast cells

Iron deficiency *per se* also has a profound impact on other immune cells, which, in children in the first stage, is associated with Th1-associated cytokines such as IL6, TNFα, and IFN-γ inflammation (96) and, in later stages of more severe cases of iron-deficient anemia, this is associated with the Th2-associated cytokine IL4 (96–98). The reason for this shift of Th1-associated cytokines toward a Th2 milieu is the fact that Th1 cells are particularly sensitive to iron deprivation (99), with the result that under iron-deficient conditions, only Th2 cells are left.

Moreover, the antibody-producing B cells, though quite resistant to iron-deprived conditions, are primed. Under iron-deficient conditions, the activation-induced cytidine deaminase (AID), an enzyme responsible for class-switch and affinity maturation, is not repressed by ferrous iron and becomes activated (100). Thus, iron deficiency is linked to C-reactive protein (CRP) and elevated IgE levels (53, 101) irrespective of the cause (86, 102–104). Interestingly, iron fortification strategies, but not deworming, have been shown to reduce IgE levels and improve iron status in Vietnamese children (105) (NCT01665378, NCT00116493).

**FIGURE 2:**
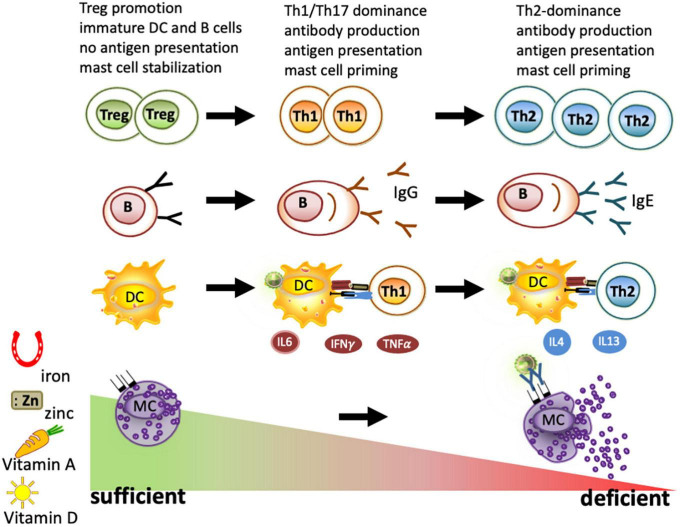
Immune cells under deficient and sufficient conditions of iron, zinc, and vitamins A and D. As part of nutritional immunity, depletion of iron, zinc, vitamin A, and vitamin D leads to immune activation and may result in a strong Th1/Th17 dominated immune response and B-cell maturation to plasma cells. When these deficiencies persist, a Th2 milieu is generated due to the higher resistance of these cells to survive under nutrient-deprived conditions. Locally, iron depletion is sufficient for mast cell priming and to evoke degranulation. In contrast, the sufficiency of iron, zinc, and vitamins A and D is known to promote regulatory T cells and keep dendritic cells, B cells, and macrophages in an immature state. All these factors have been reported to lead to mast cell stabilization, thus reducing mast cell degranulation.

Furthermore, mast cells degranulate concentration-dependently upon incubating with the iron chelator desferal (desferrioxamine) *in vitro* (106) and in the human skin (107, 108). Moreover, restricting the iron supply of mast cells by desferal activated mast cells releases inflammatory cytokines (109). Conversely, supplying mast cells with iron-saturated transferrin, lactoferrin, and beta-lactoglobulin (holoBLG) prevents mast cell degranulation (110–114) (Figure 2).

To summarize, immune cells are primed under iron-deficient conditions, which renders them hyperactive, but it also impedes iron absorption in the absence of an infection.

### 2.2. Zinc

Zinc is the third most abundant trace element and an essential component for a large number of enzymes and, as such, is crucial, particularly, in tissues that have a rapid differentiation and turnover such as the immune system, the skin, and the gastrointestinal tract.

Although important, the prevalence of zinc deficiency is uncertain due to the lack of reliable and widely accepted markers to assess zinc status. Plasma, serum, and hair zinc concentration, as well as zinc-erythrocytes, are only able to detect severe deficiencies. The WHO estimates that about 20% of the world’s population could be at risk of zinc deficiency (115), which is strongly associated with iron deficiency as both are linked to the same food sources (meat, poultry, and fish) and, in both cases, absorption is inhibited by phytates. However, in contrast to iron, zinc is not affected by blood loss and absorption is not improved by vitamin C. Bioavailability of zinc is dependent on the dietary composition with zinc sulfate and zinc acetate allowing absorption (116), while zinc oxide and zinc carbonate are insoluble and result in poorer absorption. Reported zinc bioavailability ranges from 15 to 92% (117) with phytates and dietary calcium inhibiting its bioavailability (but not phenolic compounds as with iron), while protein and citrate improve it (118). Competitive interactions can occur between zinc and other trace elements such as iron and copper, but only when present in large amounts.

The main regulatory mechanisms for zinc homeostasis in humans are absorption and excretion, with zinc being absorbed in the duodenum and jejunum (118) and mainly excreted *via* the stool (116).

Total zinc content in the human body is about 2–4 g, with 59% of all zinc contained in the muscles, 29% in the bones, 6% in the skin, 5% in the liver, 1.5% in the brain, 0.7% in the kidneys, 0.4% in the heart, and 0.1% in the hair as well as plasma.

Clinical manifestations of zinc deficiency are largely unspecific, but severe deficiencies are associated with symptoms of dermatitis, lymphopenia, retarded growth, mental disturbances, and recurrent infections (115).

#### 2.2.1. Zinc under inflammatory conditions

Similarly, as with iron, serum zinc levels decline during inflammation as a response to nutritional immunity and uptake in the liver (119), with hepcidin also blocking dietary zinc uptake *via* ferroportin-independent mechanisms that lead to downregulation of the zinc-exporter ZnT1 (120). In the circulation, 70% of zinc is bound by albumin, which is a negative acute phase protein and thus also decreases under inflammatory conditions. Importantly, and similar to iron, zinc supplementation during the acute phase or an infection may be harmful and even aggravate inflammation (24).

#### 2.2.2. Zinc immune function

On the molecular level, some functions of zinc have been linked to its role as a second messenger in immune cells with changes in the intracellular-free zinc concentration induced by the binding of various ligands to their respective receptors, e.g., Toll-like receptor 4 (TLR-4), or crosslinked immunoglobulin E bound on the high-affinity immunoglobulin E-receptor (FcεRI) (121, 122). T-cell maturation depends on zinc since zinc alone is able to promote regulatory T cells and Th1 responses *in vitro* (123, 124). In antigen-presenting cells, stimulation by lipopolysaccharide (LPS) leads to intracellular zinc mobilization from lysosomes (125) and export, which is associated with the generation of pro-inflammatory cytokines in monocytes (126) and the upregulation of major histocompatibility complex (MHC) class II molecules (127), formation of neutrophil extracellular traps by neutrophil granulocytes (33), or proliferation of T cells (34). Therefore, zinc deficiency has been associated with abrogating oral tolerance and fostering mucosal inflammation (128) (Figure 2).

#### 2.2.3. Zinc in atopy

In studies assessing trace elements, zinc deficiency is not associated with atopy in children (129–132) (NCT03408275, NCT03407391, NCT03269253, and ACTRN12606000281594) nor does maternal zinc intake reduce the risk of wheeze and eczema in the offspring (133). However, maternal intake of zinc during pregnancy is associated with better lung function in the offspring (134) (NCT03408275, NCT03407391, and NCT03269253) and lower odds ratio for wheezing during childhood, but not with atopic diseases or asthma (135). In a Polish study (NCT01861548), higher zinc and copper concentrations in cord blood were associated with an increased likelihood of wheezing in 1-year-old children affected by second-hand smoking, but not in others. No associations were determined with the levels of vitamins A and E (131, 136, 137) (NCT01861548). Still, low levels of zinc in serum, hair, and erythrocytes are consistently reported in subjects affected by atopic dermatitis (138, 139) (RMC 14193/14 and HREC 473/2017), with zinc transporter (140) and zinc-dependent enzymes being decreased in atopic lesions (141). However, zinc levels do not change with the severity of the disease (142). Also, in patients suffering from atopic asthma, low zinc levels are associated with total IgE levels (143), and a meta-analysis associated decreased zinc and selenium levels with an increased risk of asthma (144).

Taken together, zinc is a very important trace element, the bioavailability of which is limited under inflammatory conditions. Although not directly associated with the onset of allergies, its availability declines in atopic individuals presumably due to low-grade inflammation.

### 2.3. Vitamin A

**FIGURE 3:**
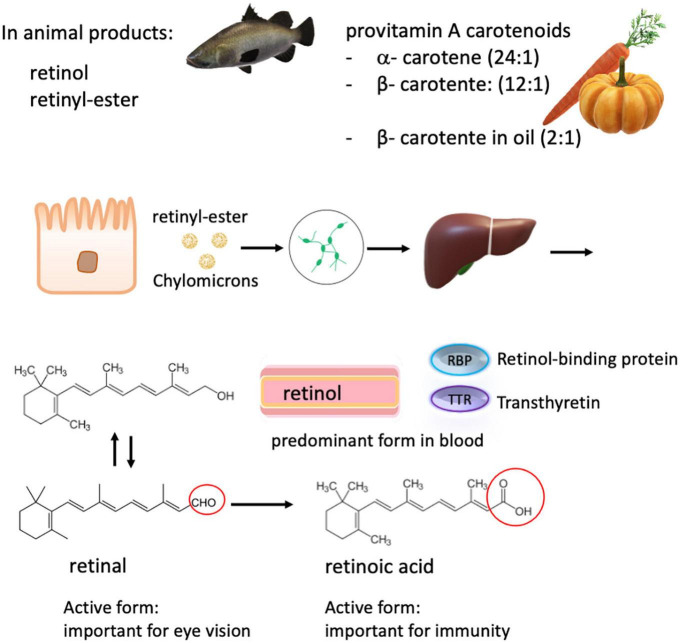
Carotenoids and retinoids metabolism. Total vitamin A intake consists of many dietary forms including free retinol, retinyl esters from animal sources, and plant-derived α- and β- carotenes, which have a lower bioavailability. The conversion rate of carotenes to retinol is in brackets. The various ingested forms of vitamin A are then processed, stored in the liver, and can be released into the systemic circulation on demand. Retinol is the major form present in the blood, which is transported by retinol-binding proteins (RBP) to the target tissue. In the circulation, RBP binds to transthyretin, probably to evade loss by glomerular filtration. Retinol is converted into retinal and retinoic acid, respectively. Retinoic acid is responsible for most of the activity of vitamin A, except for visual pigment effects that require retinal.

Vitamin A is a fat-soluble vitamin, which includes retinol, retinal, retinoic acid, and several provitamin A carotenoids. *Via* diet, preformed vitamin A (retinol) in animal food sources presents the best source as it can readily be used by the body, while provitamin A carotenoids derived from vegetables and fruits have to be converted into retinol by tissues such as the intestinal mucosa and the liver. The conversion rate of β-carotene to retinol is approximately 12:1, which is better than for other provitamin A carotenoids with a conversion rate of 24:1 (115, 145, 146) (Figure 3). The addition of oil can, however, improve the absorption of food carotenoids as well as food processing such as cooking and grinding (147). Vitamin A supplements often use synthetic β-carotene in oil with a conversion rate of 2:1 to retinol, and the synthetic forms of β-carotene in fortified foods have a conversion rate of 6:1. (93). Uptake of retinoids and provitamins begins in the intestinal lumen, which is converted into retinyl esters and is transported to the liver *via* the lymphatic system. In contrast, retinol transport is thought to occur predominantly through the bloodstream before delivery to target tissues, such as the retina (148, 149) (Figure 3).

Retinol is the predominant circulating form of vitamin A in the blood. In response to tissue demand, it is released from the liver in a 1:1 ratio with its carrier protein, retinol-binding protein. There, this complex can combine with transthyretin (150). Specific receptors on target cell surfaces or nuclei bind this complex or its active metabolites, thereby regulating many critical functions in the body, including vision, epithelial tissue integrity, and immunity (Figure 3). As zinc is required for the synthesis of retinol-binding protein, zinc deficiency reduces the amount of circulating retinol, causing a functional vitamin A deficiency, even when liver stores may be sufficient.

Vitamin A is essential for regulating embryo development and growth and for chromophore synthesis in the eyes and is an equally important nutrient for the immune system (151, 152). Vitamin A distribution takes place mainly in two ways within the body: the extrinsic pathway transports dietary vitamin A in lipoproteins *via* chylomicrons from intestinal enterocytes to tissues, while the intrinsic pathway distributes vitamin A from hepatic stores bound to the serum retinol-binding protein (RBP) (153) (Figure 3).

Vitamin A deficiency can manifest in clinical ocular signs such as night blindness and xerophthalmia, but otherwise is largely non-specific (115). The WHO estimates that about 250 million preschool-aged children throughout the world have subclinical or clinically relevant low serum vitamin A levels, and vitamin A supplementation likely reduced all-cause mortality (154, 155).

Subclinical vitamin A deficiency is linked to a worsened outcome and disease course and is closely linked to iron deficiency and to inflammation (156–158). Retinol is mobilized from the liver by an iron-dependent enzyme (159), with iron-deficient conditions hindering the mobilization of this vitamin. Importantly, retinol supplementation during infancy did not increase the risk of atopy at age 7 (160) (NCT00168597 and NCT00168584). In contrast, retinol deficiency aggravates asthma (161), allergic rhinitis (162), and atopic dermatitis (163).

Retinol is also closely linked to the immune system. In the case of any infection or inflammatory process, serum retinol declines (164, 165), and this decline is associated with an increase in CRP (166). Therefore, in the presence of inflammation, this can result in an overestimation of vitamin A deficiency.

#### 2.3.1. Impact of vitamin A deficiency on an immune cellular level

It has been reported that vitamin A deficiency leads to a shift toward Th1, giving rise to the production of IFNγ (167, 168) and negatively affecting the antibody response. In line, vitamin A deficiency also hindered the conversion of inflammatory monocytes into tissue-resident macrophages. Thereby, the resolution of type 2 inflammation as well as the removal of the infectious agent was hindered which led to increased mortality *in vivo* (169). Similarly, the study by Rühl et al. demonstrated that a retinoic acid-deficient diet promotes the Th2-associated IL4 as well as IFNγ in a mouse model (170) and led to elevated IgE antibody production (171). Another study showed that oral tolerance was impaired in retinoic acid-deficient mice (172) and that oral supplementation with carotenoids inhibited oral sensitization and food allergy (173, 174).

Retinoic acid can antagonize the development of innate lymphoid cell (ILC)2s while promoting the expansion of ILC3s and imprinting DCs with the ability to produce retinoic acid, thereby inducing naïve T cells to differentiate into T-regulatory cells (175). In addition, inhibition of NF-κB signaling by retinoic acid in macrophages has been described. Retinoic acid is able to suppress the differentiation of Th1 and Th17 cells, enhance regulatory T cells, and inhibit proliferation and differentiation of B cells, and reduced mediator release from mast cells was reported after retinoic acid treatment *in vivo* (151, 176, 177) (Figure 2).

### 2.4. Vitamin D

**FIGURE 4:**
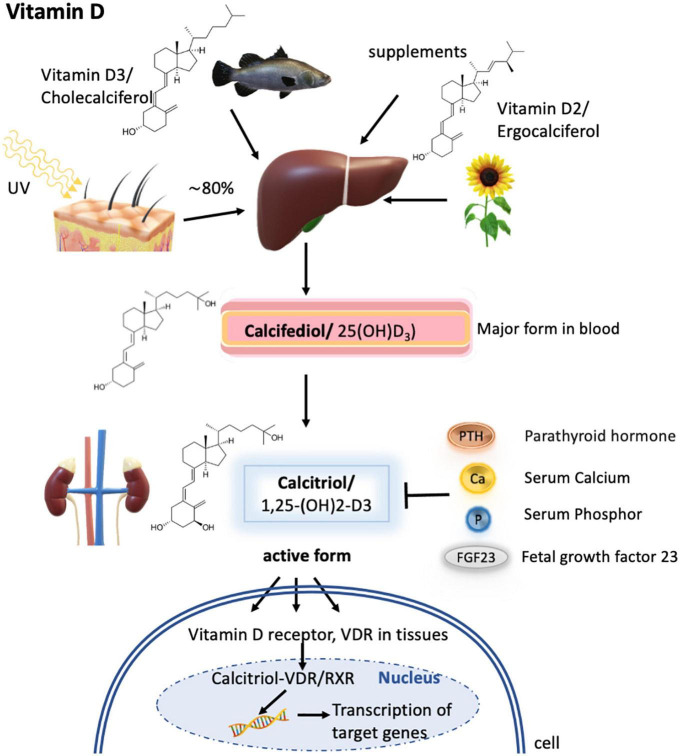
Vitamin D metabolism. Vitamin D3 is synthesized mainly in the skin by UV radiation, but a small fraction can also be obtained *via* diet as vitamin D3 or vitamin D2. In the liver, both forms are metabolized to calcifediol (by CYP27A1), which is the major form present in the blood but is not biologically active. Only upon further hydroxylation in the kidney, calcifediol is turned into its active form termed calcitriol. Calcitriol synthesis is known to depend on calcitriol itself, the parathyroid hormone, serum calcium concentrations, serum phosphor concentrations, and fetal growth factor 23. The active form then subsequently binds to the vitamin D receptor (VDR) expressed in different tissues, which then enters the nucleus to form heterodimers with the retinoid X receptor/RXR and activates the transcription of calcitriol target genes.

Vitamin D is a fat-soluble vitamin and a key regulator for calcium and phosphorus homeostasis. It regulates cell differentiation and hormones such as parathyroid hormone and insulin. About 80% of vitamin D (calciferol) is synthesized in the skin of most animals, including humans, from its precursor, 7-dehydrocholesterol, by ultraviolet light exposure from sunlight (Figure 4). This produces a naturally occurring form of the vitamin known as vitamin D3. Vitamin D is naturally present in relatively few foods; thus, a small fraction of the daily requirements for the vitamin is supplied through diet. Salt-water fish, such as herring, salmon, sardines, and fish liver oil, is a rich source of vitamin D3, whereas plant analogs are known as vitamin D2, which has, however, about a third of the activity of vitamin D3 (178, 179). Small quantities of vitamin D are found in other animal products (e.g., beef, butter), and if hens are fed a vitamin D diet, eggs can provide substantial amounts of the vitamin (115).

Several studies have shown that the effects of poor vitamin D status are exacerbated by low calcium intakes, also in children (180) (NCT00949832).

Metabolic processing of vitamins D3 and D2 is similar, with the first metabolization step occurring in the liver to 25-hydroxy vitamin D (25-OH-D3, also termed calcifediol). Calcifediol is the major form present in blood and its concentration determines how much will be transported to the kidney and metabolized into 1,25-dihydroxy vitamin D [1,25-(OH)2-D3, also called calcitriol]. Calcitriol is the biologically active form of the vitamin and only this form can act *via* the vitamin D receptor (VDR) expressed on different tissues. Upon calcitriol-binding, the VDR enters the nucleus in the cell and forms heterodimers with the retinoid X receptor/RXR for DNA binding and activation of transcription of the calcitriol-responsive genes (181). Transport of vitamin D_3_ and its metabolites to the kidney and target organs occurs *via* plasma vitamin D binding protein (DBP) (179) (Figure 4). It has to be emphasized that the retinoid X receptor is essential for the transcription of vitamin D3-responsive genes, but is also a transcription factor for retinoic acid. Therefore, vitamin A can be a regulatory element in the biological action of vitamin D.

Calcitriol, the active form of vitamin D, is controlled by its production, by the parathyroid hormone, which stimulates the renal production of calcitriol; fetal growth factor 23; and serum levels of calcium and phosphate (179) (Figure 4).

Severe vitamin D deficiency results in a bone disease called rickets in infants and children, and osteomalacia in adults, which are characterized by the failure of the organic bone matrix to calcify. The lesions are reversible after the correction of vitamin D deficiency. The global prevalence of vitamin D deficiency is uncertain, but it is greater in those living at high latitudes where daylight hours are limited in the winter months, in those with darker skin due to reduced capacity to produce vitamin D and where clothing inhibits UV radiation from the sunlight (182).

As vitamin D greatly influences bone homeostasis, which is also a major and essential site of immune cells, deficiencies of this important vitamin greatly impact our immune system (151, 152). Also here, deficiencies are associated with inflammation and disease severity (183, 184) in several immune-driven diseases with chronic inflammation (185, 186).

Vitamin D can also suppress hepcidin and, therefore, promote dietary iron uptake (187), but vitamin D deficiencies also facilitate iron deficiency.

#### 2.4.1. Impact of vitamin D deficiency on an immune cellular level

Calcitriol is reported to enhance chemotaxis and antimicrobial peptide synthesis in monocytes and macrophages and is involved in the activation of type 3 innate lymphoid cells (ILC3s). Vitamin D3 inhibits the maturation of DCs, thereby leading to a tolerogenic state with low antigen presentation and increased IL-10 production. It also regulates and suppresses IL2-production in activated T and B cells (188, 189). Moreover, the differentiation of Th1 and Th17 cells is hindered, whereas regulatory T-cell generation is promoted and the proliferation and differentiation of B cells are inhibited. It has been reported that vitamin D3 contributes to stabilizing mast cells and reducing mediator release from effector cells after retinoic acid or vitamin D3 treatment *in vivo* (151, 176, 177) (Figure 2).

### 2.5. Prevalence of vitamin A and vitamin D deficiency in children with atopic diseases

Atopic diseases in children include atopic dermatitis, rhinitis, asthma, and food allergy (190). As allergic diseases can be influenced by micronutrients such as vitamins A and D, many studies have looked at serum vitamin levels in the allergic pediatric group and investigated the effects of vitamin supplementation (191, 192).

In this respect, two recent studies showed lower serum vitamin D and retinol levels in children affected by atopic dermatitis compared to healthy controls (193, 194). In addition, the retinoid-mediated signaling in the skin seemed impaired. Increased severity of atopic dermatitis was associated with significantly lower levels of serum vitamin D, which was also reported by others (195, 196) (CRD42017068773). Children with asthma have lower circulating vitamin A levels (197), which could be ameliorated by dietary means (198). Besides, the intake of carotenoids, beta-cryptoxanthin, and alpha-carotene was inversely associated with allergic skin sensitization (199). Vitamin A deficiency in infancy and early childhood was also associated with the subsequent development of allergies (200).

For food allergy, the number of studies on vitamin A is limited, while vitamin D is more prominent in recent investigations. A case–control study revealed that children with food allergy had lower vitamin D levels compared to healthy controls and were more likely to suffer from multiple food allergies (201). Another study showed that micronutrient intake of this precious nutrient was lower in children affected with food allergies (202). In a large retrospective study including children with food allergy and other atopic conditions, vitamin D deficiency was detected in one-third of all children, and an association of food allergy to lower iron and transferrin saturation levels and a higher eosinophil percentage were described (203). The role of vitamin D in food allergy is still controversial as other studies have shown no association of vitamin D in the development of food allergy (204). However, since genetic variation concerning vitamin DBP could also influence serum vitamin D levels in food allergy (205) as well as in atopic dermatitis (206), this should be further explored.

A recent case–control study in Chinese children with stable asthma reported significantly decreased serum vitamin A and D levels in the asthmatic group, with a positive correlation of vitamin levels to good pulmonary function and quality of life (207). The findings of reduced serum vitamin A and D levels in asthmatic children compared to healthy controls have been known for some time (208) and are supported by recent studies. Andino et al. demonstrated it for serum vitamin A in a small cross-sectional case–control study, and Omole et al. demonstrated it in a larger comparative cross-sectional study for serum vitamin D (209, 210). In addition, low serum vitamin D levels (<20 ng/ml) in asthmatic children seem to be associated with a higher serum eosinophil count and total IgE, thereby correlating with the severity of childhood asthma (211, 212).

Other birth-cohort studies correlate maternal intake of vitamins A and D to asthma or allergy risk in the offspring. Maternal vitamin D sufficiency throughout pregnancy attenuated the risk of recurrent wheezing or asthma in the offspring up to 6 years of age and especially in children with asthmatic mothers (213). In a randomized placebo-controlled trial, high-dose vitamin D supplementation in mothers significantly decreased the risk of allergic rhinitis and allergic sensitization in the offspring at 6 years of age (214) (NCT00920621), but not the risk of developing asthma (215) (NCT00920621). Parr et al. reported an increased asthma risk of 7-year-old children after excess maternal dietary intake of vitamin A, while vitamin D intake close to recommendations reduced the childhood asthma risk (216) (NCT03197233).

In summary, vitamin A and D deficiencies are common in children affected by atopic diseases. However, data on prenatal and childhood vitamin A and D supplementation demonstrate the difficulty in correlating dietary/supplementary vitamin intake with allergic diseases and the need for further observational and intervention studies, especially for vitamin A, in consideration of dosage, timing, and type of supplementation. Nevertheless, vitamins A and D and their metabolites are essential, key components for the development and homeostasis of the immune system, and as important nutrients in the human diet, they influence oxidative stress and inflammation, two central factors in the clinical manifestation of allergic diseases (208).

## 3. Dietary intervention for disease prevention and amelioration of the disease course

### 3.1. Whole food to ameliorate atopy

Regarding atopic diseases, it is clear that diet can prevent the disease course. Consumption of fruits and vegetables is known to improve asthma control and the risk of exacerbation in adults (217) (ACTRN012606000286549). Although this could not be reproduced by the same study group in children (218) (ACTRN12615000851561), another study observed a beneficial effect (219) (ACTRN12615000851561). A meta-analysis confirmed that the consumption of vitamins A, D, and E; zinc; fruits and vegetables; and a Mediterranean diet protected against asthma (220).

Maternal intake of foods commonly considered allergenic (peanut and milk) was associated with a decrease in allergy and asthma in the offspring of a pre-birth US cohort (221). Similarly, maternal fish and apple consumption were found to be protective against the onset of asthma (222). Furthermore, a large Danish National Birth Cohort associated the ingestion of peanuts, tree nuts (223), and/or fish (224) during pregnancy with a decreased risk of asthma. However, epidemiological studies and randomized controlled trials (RCTs) on maternal intake of fish oil (not fish) did not reduce atopy in children aged 6 years (225), whereas long-chain polyunsaturated fatty acids supplementation during pregnancy showed no significant impact on atopy (226, 227) (ACTRN12605000569606, ACTRN12610000735055, and ACTRN12615000498594) and only a small effect on the risk of asthma (228) (NCT01353807). A systematic review by Venter et al. associated the maternal consumption of vegetables and yogurt with the prevention of any allergy (229). Furthermore, lower maternal egg intake was associated with higher serum total IgE and peripheral eosinophilia in children with atopic dermatitis (230).

Whereas maternal intake already has an impact on the development of allergic diseases, studies consistently highlight the impact of a healthy diet in children.

In the Spanish ISAAC phase III with over 20,000 schoolchildren, the consumption of cow’s milk, butter, and nuts was found to reduce the risk of atopic dermatitis (231), and also in the GABRIELA cohort, raw cow’s milk consumption was found to protect against asthma and atopy, with the whey protein levels being inversely associated with asthma (232). It must be stressed that foods that are considered allergenic seem to protect against the development of allergies. This dichotomy is partly explained by the fact that major allergens can bind to minerals such as iron (110, 233–235) and zinc (236), but also vitamins such as vitamins A (227) and D (27, 237, 238). Indeed, studies carried out in the last decade suggest that this feature is an important aspect of atopy preventive effects. In this respect, it has been demonstrated that proteins carrying micronutrients provide those *via* the lymph to the immune system and promote tolerance. In contrast, when the same proteins did not carry these micronutrients, they turned into allergens (110, 114, 233–235, 237, 239–242).

Moreover, in a small study of children with atopic asthma, consumption of a whey-based oral supplement for a month reduced IgE antibodies and improved lung function (243). It is important to note that the beneficial impact of milk to prevent atopic diseases correlates with the whey protein levels and is lost in cooked milk (232, 244–246). In an RCT from Brazil, it was found that milk beverages fortified with micronutrients and prebiotics for 6 months decreased the risk of allergic manifestations by 36% (NCT01431469) (247).

In another clinical trial, whey spiked with micronutrients was packed in a lozenge to exploit specifically beta-lactoglobulin as a carrier for iron, vitamin A, and zinc, in order to bring these micronutrients *via* the lymph system to the immune cells. Consumption of this holoBLG lozenge for 3 months led to an amelioration of allergic symptoms in patients with allergic rhinitis to house dust mites, reducing total nasal symptoms by 60%, and they reported greater perceived wellbeing measurable even up to 8 months after cessation of holoBLG supplementation (248, 249) (NCT04477382, NCT04872868, and NCT05455749). Similarly, also in a double-blind, placebo-controlled pilot trial, 6-month supplementation with this holo-BLG lozenge significantly ameliorated symptoms by about 40% over placebo in grass and birch pollen allergic women (45) (NCT03816800). In line with the beneficial impact of dietary intake of heat-sensitive whey proteins, in a human pilot study, it was found that drinking raw milk is tolerated better in allergic children than highly processed shop milk (250) (Bo/06/2009).

Taken together, the consumption of food that is particularly considered allergenic early in life such as milk, whey products, fish, nuts, fruits, and vegetables, is beneficial to prevent and ameliorate atopic diseases.

### 3.2. Iron to ameliorate atopy

Although iron deficiency is highly prevalent in atopic diseases, and there are national strategies in place to combat iron deficiency and anemia by dietary means, very few studies have analyzed the impact of iron supplementation in atopic diseases and the few available foci on the perinatal period. In the EDEN cohort, based on a food questionnaire, a high maternal intake of red meat (more than three to four times per week) in the year preceding pregnancy was associated with a risk of wheezing, whereas no association was found during pregnancy (251). In the study by Fortes et al. (89), maternal supplementation of iron and folic acid during pregnancy decreased the likelihood of their offspring developing atopic dermatitis by 80%. In a follow-up study of a population-based, multicenter, RCT, maternal iron supplementation regardless of hemoglobin levels, was also associated with a reduction in the risk of asthma in the offspring by 42% and nearly 70% in the offspring of asthmatic mothers (252). In a double-blind, placebo-controlled pilot trial conducted in birch and grass pollen allergic in women, in which the immune cells were supplemented with iron, a 40% amelioration of symptoms was reported (45).

No intervention study with iron supplements or fortified foods has analyzed the outcome of atopic diseases and asthma in children so far.

As many studies are reporting a protective impact of fish as well as vegetable and fruit intake, in preventing atopy, the right form and quantity of iron intake may be an important aspect. Fish is a rich source of iron, but also omega-3 fatty acids and an adequate intake of vitamin C (ascorbic acid) from fruits and vegetables facilitate iron uptake, which leads to consideration of the protective impact of iron *via* these food sources. As many children not only are iron-deficient but also lack vitamin A, their iron status can also be ameliorated by incorporating vitamin A into their diet. Indeed, it has been demonstrated that in adolescent girls, co-supplementation of oral iron with vitamin A improved the uptake and efficacy of iron supplementation in the presence of low-grade inflammation (253) (NCT 01198574).

### 3.3. Zinc to ameliorate atopy

In line with the uncertainty of zinc in the onset of atopy, no association of maternal zinc intake with atopy has been established (131). However, as zinc deficiency is a common finding in children affected by atopic diseases, zinc supplementation for 8 weeks in zinc-deficient children with moderate asthma and on inhaled steroids significantly improved their clinical symptoms and lung function but not total IgE levels (254). Conflicting results have been obtained on children with atopic dermatitis. While in children with atopic dermatitis, 8-week oral supplementation with zinc improved eczema severity and hair zinc levels compared to a non-supplemented control group (255), but another placebo-controlled trial did not observe any beneficial effect (256).

### 3.4. Vitamin A to ameliorate atopy

Only a few studies have been carried out on vitamin A showing that dietary intake of beta-carotene is associated with a reduced risk of allergic sensitization and lower IgE levels, in 5- and 8-year-old children (257) and women (132) (NCT03408275, NCT03407391, NCT03269253, and ACTRN12606000281594). However, in one study, ß-carotene intake was associated with an increased risk of hay fever in adults (258) (SRCTN72673620). Another study showed that high-dose supplementation of vitamin A in infants in Guinea-Bissau, an endemic vitamin A deficient region in West Africa, did not increase the risk of atopy (160) (NCT00168597 and NCT01779180). Neonatal high-dose vitamin A supplementation did not increase the overall risk of atopy; however, the female gender was at greater risk of atopy and wheezing (23) (NCT01779180). It should be pointed out that in this study, the infants were given 50,000 IU of highly bioavailable retinyl palmitate, and adverse effects of hypervitaminosis A were reported, occurring with intakes as low as 1,500 IU/kg in vulnerable groups such as children (259). In this respect, one might wonder whether the given dose tended to be too high for female infants who tend to be smaller in height and weigh less than boys of the same age.

In another study conducted in an area with chronic vitamin A deficiency, vitamin A supplementation was not associated with an increased prevalence of asthma (260). Dietary intake of vitamin A was evaluated in 7-year-old children in a population-based birth cohort in the UK together with lung function and asthma risk and revealed that high dietary preformed vitamin A, but not ß-carotene intake, was associated with higher lung function and lower incident asthma risk (261) (NCT03408275).

Interestingly, an earlier prospective birth cohort study found that supplementation of children in the first year of life with vitamins A and D in the water-soluble form increased the risk of food allergy and asthma twofold at the age of 4 years, compared to children receiving the same formulation in oil suspension (262). In this respect, it must be noted that the difference in vitamin adsorption in the intestine depends on the vitamin formulation, as lipid-soluble vitamins A and D are incorporated into chylomicrons together with other lipid metabolites and enter the general circulation mainly *via* the lymphatic pathway (263, 264), whereas vitamins A and D in water-soluble form do not seem to take the lymphatic pathway.

In the Korean version of the International Study of Asthma and Allergies in Childhood (ISAAC), a reduced atopic dermatitis risk was associated with elevated serum retinol levels (60).

In a meta-analysis, oral supplementation with vitamin D, combined vitamins D and E, combined vitamins A, D, and E, and topical vitamin B_12_ was associated with a significantly lower severity score for atopic dermatitis (265).

Therefore, although there is evidence that food containing vitamin A seems to prevent atopic diseases, the form of vitamin A seems to be essential for bioavailability and very likely explains the conflicting results obtained in the different studies.

### 3.5. Vitamin D to ameliorate atopy

Since vitamin D is predominantly produced *via* the skin, analyzing dietary vitamin D intervention studies is quite challenging as sun radiation should be taken into consideration. Indeed, sun exposure has also been linked to protection from and reduced asthma prevalence in schoolchildren (266). Moreover, in a randomized, controlled trial, vitamin D supplementation improved winter-related atopic dermatitis in Mongolian children (267) (NCT00879424).

A systematic review of cohort, case–control, and cross-sectional studies concluded that maternal dietary intake of vitamins D and E is associated with a lower risk of wheezing illnesses in children, with another concluding that prenatal vitamin D supplementation may reduce the risk of asthma in the offspring (268). However, higher rates of cow’s milk allergy, but not respiratory allergies, were observed in a Finnish intervention study in infants who were given 1,200 IU of vitamin D daily compared to 400 IU for 1 year, which was associated with higher blood vitamin D3 levels (NCT01723852) (269) and highlights that only deficiencies should be treated as an excess of these micronutrients may also cause inflammation and thereby promote sensitization. As calcium is essential for the conversion of vitamin D3 into calcitriol, the question arises as to whether the diet of these children contained enough calcium for conversion, particularly, as cow’s milk, which is rich in calcium, was likely excluded from their diet.

Maternal vitamin D supplementation in standard vs. high dose during pregnancy could not confirm nor rule out a protective effect (270, 271) (NCT00856947 and NCT00920621), whereas a combination of the two trials reported a 25% reduction of the risk of asthma/recurrent wheeze (272, 273). However, perinatal supplementation was not sufficient to influence the 6-year incidence of asthma and recurrent wheeze among children who were at risk of asthma (215) (NCT00920621).

In a randomized placebo-controlled trial, high-dose vitamin D supplementation in mothers significantly decreased the risk of allergic rhinitis and allergic sensitization in the offspring at 6 years of age (214) (NCT00920621), but not the risk of developing asthma (215) (NCT00920621). Parr et al. reported an increased asthma risk of 7-year-old children after excess maternal dietary intake of vitamin A, while vitamin D intake close to the recommended dose reduced the childhood asthma risk (216) (NCT03197233).

The results of vitamin D supplementation during childhood up to 18 years differed in the latter studies: in both studies, vitamin D supplement was given for 3 months in a similar dosage, leading to reduced atopic dermatitis symptoms in the pre-post interventional study by Imoto et al. (195) (CRD42017068773), while no change in severity compared to placebo was found in the randomized-controlled trial by Lara-Corrales et al. (196).

To sum up, there are conflicting data on prenatal and childhood vitamin D supplementation that demonstrate the difficulty of correlating dietary/supplementary vitamin intake with allergic diseases, and further studies are needed that take dosage, timing, type of supplementation, as well as sun exposure into consideration. Nevertheless, D vitamins and their metabolites are key players in immune homeostasis and are important nutrients in the human diet that could influence oxidative stress and inflammation, two central factors in the clinical manifestation of allergic diseases (208).

## 4. Discussion

Micronutrients are pivotal and, as such, many of these deficiencies can be prevented through nutritional education, the consumption of a healthy, varied diet, as well as by fortifying and supplementing foods as needed.

In particular, a diet that is low in animal-source foods typically results in low intakes of bioavailable iron and zinc, calcium, retinol, vitamin B2 (riboflavin), vitamin B6, and vitamin B12 (274). Poor quality diets often lack fresh fruits and vegetables resulting in insufficient intakes of essential micronutrients such as vitamin C (ascorbic acid), carotene (provitamin A), and folate. Another important aspect is that the milling of cereals also removes several nutrients, notably, iron and zinc, various B vitamins (i.e., thiamine, riboflavin, and niacin), and folate (275).

Moreover, the quality of breast milk differs greatly. The breast milk of undernourished lactating women, who have an underlying low-grade inflammation present and/or consume a limited range of foods and are thus affected by multiple micronutrient deficiencies, is most likely to have low levels of vitamin A (retinol), iron, the B vitamins, iodine, and selenium (18–21). An important issue is also that breast milk alone is no longer sufficient to meet the nutritional requirements in terms of energy and micronutrients (iron and zinc) after 6 months of age. The European Food Safety Authority concluded in a 2019 systematic literature search that as long as foods have an age-appropriate texture, no adverse effects on health are associated with complementary feeding. The majority of infants need complementary food from around 6 months of age, and children at risk of iron depletion, in particular, may benefit from an earlier introduction of complementary food (276). As several studies suggest that milk from atopic mothers differs in its composition and nutrient content, complementary feeding and a diverse diet for the mother should be encouraged.

Micronutrients are essential not only for proper growth but also for a healthy immune system. Indeed, studies consistently reveal that children with atopic diseases are strongly affected by micronutritional deficiencies which are due, on the one hand, to an inadequate intake of these micronutrients and, on the other hand, due to inflammation that further impairs specifically iron and zinc uptake.

Clinicians and nutritionists should be more aware of the fact that first, functional iron deficiency in people with underlying inflammatory diseases will negatively affect the disease course, and second, the normal absorption of these important trace elements is inhibited or impeded so that alternative strategies for meeting the demands are necessary. Some studies have reported a relatively high prevalence of parasitemia in children with allergic diseases. As there is some evidence that these may aggravate the disease course (65, 277–279), patient management should include deworming strategies when required.

Deficiencies in iron, vitamin A, and vitamin D facilitate inflammation as they render the immune system hyperactive, which is prominently displayed in the case of anemia of inflammation. In atopic diseases, the overdrive of the immune system poses a particular problem, as there is usually no infectious agent present. Yet the absorption of iron and zinc and likely also of vitamins from the diet is hampered. We highlight these very important aspects in Table 1, which should be considered when conducting nutritional clinical studies.

**TABLE 1:** Important aspects for clinical nutritional studies.

**General**
• Blood analysis after fasting • Strong circadian rhythm known for iron, Vitamin A and D • Micronutrient-levels decline under inflammatory conditions • Parasitic infections should be excluded
**Iron**
• Assessment of iron parameters • Measurements of inflammatory markers • Improved bioavailability with Vitamin C and Vitamin A • Improved bioavailability by addition of whey and Vitamin A under inflammatory conditions
**Zinc**
• Assessment of zinc parameters • Measurements of inflammatory markers • Decreased under inflammatory conditions • Improved bioavailability by co-supplementation with albumin proteins and citrate
**Vitamin A**
• Assessment of Vitamin A • Measurements of inflammatory marker • Decreased under inflammatory conditions, affected by zinc-deficiency • Conversion rate of beta-carotenes is low without the addition of oil
**Vitamin D**
• Assessment of Vitamin D metabolites • Sun exposure has to be factored in to assess the efficacy of nutritional Vitamin D supplementation studies, which should be conducted preferably during winter-time • Measurements of inflammatory markers along Vitamin D • Calcium-rich diet along Vitamin D supplementation

The different cohorts as well as the form of food or supplementation may explain the discrepancies observed in some of the studies, particularly with vitamins. Therefore, the consumption of whole food items rather than purified food components should be encouraged as well as combining the different food sources to adequately meet the micronutrient requirements in children. Increasing dietary diversity, therefore, ensures that both the quantity as well as the range of micronutrient-rich foods are consumed (280) in a manner that ensures uptake and improves allergy outcomes. Similarly, the early introduction of “allergenic foods” such as dairy products, fish, vegetables, and fruits should be encouraged as breastmilk alone is not sufficient to meet the micronutritional demands of growing infants after 6 months.

For children suffering from atopic diseases, strategies to circumvent the mucosal block by combining different food sources and including dietary food items with a protective impact should be implemented.

In conclusion, adequate assessment and dietary management are pivotal for children with underlying chronic diseases and special attention should be given to micronutritional deficiencies as drivers of inflammation.

## Author contributions

DP conceptualized the topic, contributed to the writing, and data acquisition. KH contributed to the writing and data acquisition. PC contributed to the interpretation of data and critically revised the manuscript for important intellectual content. FR-W wrote the manuscript and prepared the figures. All authors approved the final version of the submitted manuscript.
